# Optimal acquisition parameter selection for CT simulators in radiation oncology

**DOI:** 10.1120/jacmp.v9i4.2878

**Published:** 2008-11-11

**Authors:** Ruijie Rachel Liu, Karl L Prado, Dianna Cody

**Affiliations:** ^1^ Department of Radiation Physics The University of Texas M. D. Anderson Cancer Center Houston TX U.S.A.; ^2^ Department of Imaging Physics The University of Texas M. D. Anderson Cancer Center Houston TX U.S.A.

**Keywords:** CT protocol, helical artifact, pitch, detector configuration, multichannel CT

## Abstract

The purpose of this study was to identify optimal CT acquisition parameter settings for each make and model of scanners used in a large Radiation Oncology (RO) Department, considering the special requirements of CT simulation. Two CT phantoms were used to evaluate the image quality of the five different multichannel CT scanners using helical scan mode. We compared the effects of various pitch, detector configurations, and rotation time parameters on image artifacts, and on spatial and contrast resolution. We found that helical artifact was closely related to pitch and detector configuration settings. This artifact was scanner‐specific and generally more obvious when the channel width or detector collimation was equal to the image thickness. Different acquisition parameter settings produced slight differences in observed high‐ and low‐contrast resolution. Short rotation time degraded image quality for certain scanners, but only slightly, while other rotation times, such as 0.75 sec/rotation and above, had no obvious effect on resolution. An optimized combination of acquisition parameters was determined for each scanner make and model, based on phantom image quality and other considerations for clinical applications. This information may be directly useful for physicists whose CT simulation scanners match one of the five examined in this study. If not, the strategy reported here may be used as a guide to perform a similar evaluation of the scanner.

PACS numbers: 87.57.Ce, 87.57.qp, 87.55.Gh, 87.57.C‐

## I. INTRODUCTION

Multi‐channel CT has been widely accepted for diagnostic applications as well as for radiotherapy virtual simulation purposes. The effect of CT acquisition parameters on image quality is important to all clinical applications, though the goals of the applications are quite different. A few studies^(^ ^1^ ^–^ ^6^ ^)^ regarding optimizing acquisition parameters for certain applications in diagnostic imaging (DI) using multi‐channel CT scanners partially addressed the effects of machine settings on image quality. However, a more thorough study is needed regarding image quality for newer scanners for diagnostic and therapeutic applications. CT technology continues to develop and there is a vast difference in acquisition parameter options between manufacturers.

Though there is some information available regarding technical details, such as image thickness for CT radiotherapy simulation,^(7)^ the optimal CT image acquisition parameter settings for radiotherapy simulation have not yet been thoroughly investigated.

The purpose of this study was to address this gap, and determine the optimal image acquisition parameter settings for conventional CT simulation protocols, including pitch (defined as couch movement per gantry rotation divided by beam width), detector configuration, and rotation time using several different CT scanners in our radiation oncology (RO) department. Routine radiation dose values were also collected in order to help understand image quality characteristics between scanners.

## II. MATERIALS AND METHODS

### A. Rationale for CT Simulation in Radiation Oncology

The implementation of CT simulators in radiation oncology (RO) is somewhat different from CT scanners in DI, though image quality is a main concern for both. Unlike the regular practice in DI, breath holding, contrast injection, variable image thickness, and gantry tilt are generally discouraged in RO. Because most patients undergoing CT simulation in RO have diagnosed cancers, and generally only one scan is used for CT simulation, radiation dose is of less concern than it is in DI. Instead, image quality has singular importance, as accuracy in RO treatment planning is particularly vulnerable to artifacts.

In order to simulate radiation treatment conditions, free or gated breathing is required during the CT scan. Fast scanning is generally not desirable because a snapshot of one breathing phase does not represent the average anatomy position during treatment delivery. A scan time of 1 to 2 minutes is considered acceptable. For CT simulation, patient skin surface must be included in the image for treatment planning. Image truncation is intolerable; therefore a larger display field of view (DFOV) is used, usually 50 cm or 65 cm. For a head scan, a body size DFOV is generally used, because part of the shoulders must be scanned as part of patient positioning for most procedures.

For CT simulation, the choice of image thickness involves balancing the conflicting considerations of the increased resolution of digitally reconstructed radiographs (DRR) obtained from thin images and increased physician effort required to contour multiple images. The standard choice of the physicians and physicists in our institution is 2.5 mm or 3 mm image thickness and 2.5 mm interval, which agrees with general recommendations.^(7)^

### B. Phantoms and Scanners

A Catphan phantom^(8)^ (The Phantom Laboratory, Salem, New York) was primarily used to evaluate image quality. Three modules were used in this study: 1) CTP528, high‐contrast resolution bar patterns; 2) CTP515, 1.0% low‐contrast objects in the Supra Slice portion; 3) CTP401, artifact evaluation. The high‐contrast resolution section of the GE CT daily QA phantom^(9)^ (GE Medical Systems, Milwaukee, Wisconsin) was used for additional artifact evaluation. The high contrast region was chosen to evaluate artifacts because objects with high‐edge gradients are more likely to produce helical artifacts. Both phantoms were positioned by suspending each from an appropriate bracket, following the manufacturers' standard setup recommendations. Five different CT scanners were evaluated:

General Electric (GE)

- LightSpeed (LS) RT (4‐channel, 80 cm bore)
- LS RT16 (16‐channel, 80 cm bore)
- LS16 (16‐channel, 70 cm bore)

Philips Medical Systems

- AcQsim CT (1‐channel, 80 cm bore)
- MX8000 (16‐channel, 70 cm bore).

### C. Evaluation Methods

Various pitch, detector configuration, and gantry rotation time (1 sec and less) settings were used to acquire images, while maintaining the following constant settings: 120 kVp, 240 effective mAs (defined as tube current (mA)×rotation time (sec) / pitch; where pitch=table movement per gantry rotation / total beam collimation), 1s rotation time unless specified otherwise, 25 cm display field of view (DFOV), standard reconstruction filter, 2.5 mm image interval, and 2.5 mm image thickness for GE or 3 mm image thickness for Philips.

On multi‐channel CT scanners, there are multiple data channels that route the signals collected on the detector surface to the reconstruction computer. These data channels can be utilized in a flexible manner to produce more than one thin image during one rotation of the gantry, or fewer thicker images during one rotation of the gantry. The combination of the number of active channels and the detector width (z‐direction) assigned to each channel is called the detector configuration. The product of these two values represents the nominal x‐ray beam width (z‐direction). In general, a combination of fine detector spacing (also called small channel width) and a wide beam width are the most desirable in CT imaging. Fine detector spacing produces a denser helical dataset from which to interpolate, and a wide beam allows for higher table speeds and faster exams.

The phantom images were evaluated from three aspects: 1) high‐contrast resolution; 2) low‐contrast resolution; and 3) the presence of helical artifacts. A single observer primarily evaluated all the images. Zoom, display window and level were adjusted to best display the image region of interest for high‐contrast and low‐contrast resolution evaluation. For low‐contrast resolution, since CTP 515 module spans 70 mm, the images with best resolution were selected for evaluation.

Three levels were used to score the presence of artifact: “Yes”, “Slight”, and “No”. “Yes” meant the artifact was clearly visible, “No” meant no artifact was present, and “Slight” meant the artifact was present in the phantom images, but it was very subtle and most likely invisible in clinical images.

To examine the effect of gantry rotation time, 1 sec and sub‐second rotations were evaluated for scanners that had these options. The fastest gantry rotation for GE RT and Philips AcQsim was 1 sec. Therefore they were not included in the evaluation of gantry rotation time effect. The single‐channel Philips AcQsim CT is limited by prolonged scan time and the effects of excessive tube heating, so only pitches greater than 1 were evaluated for this single‐channel CT.

In order to interpret results such as low‐contrast resolution, the standard annual radiation dose assessment made for each scanner was also reported on a normalized (per 100 mAs) basis. These dose evaluations were obtained by using the standard CT Dose Index (CTDI) approach.^(10)^

## III. RESULTS

### A. Artifact evaluation

**Figure 1:**
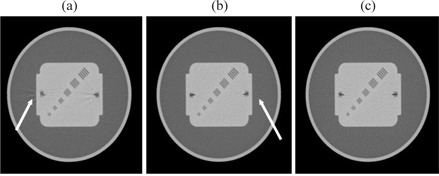
Artifacts in 2.5 mm thick images of the Daily QA phantom for GE LS16: (a) 8×2.5 mm, pitch 1.675; (b) 16×1.25 mm, pitch 1.75; (c) 16×1.25 mm, pitch 0.562.

For all CT scanners, helical artifacts were more severe when the channel width was equal to the image thickness and a relatively high pitch was used. For example, for the GE LS16 (Fig.1), a detector configuration of 8×2.5 mm and a pitch of 1.675 resulted in 2.5 mm thick images with severe artifacts Fig. 1(a), while a 16×1.25 mm, 1.75 pitch setting showed slight artifacts Fig. 1(b), and a 16×1.25 mm, 0.562 pitch had no artifacts in the images Fig. 1(c).

**Figure 2:**
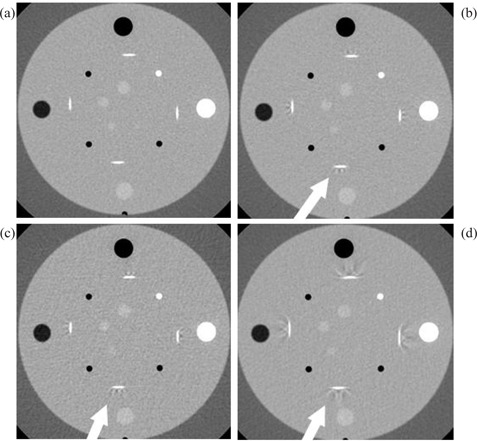
Artifacts in 2.5 mm thick images of the Catphan phantom for GE LS RT: (a) 4×1.25 mm, pitch 0.75; (b) 4×1.25 mm, pitch 1.5; (c) 4×2.5 mm, pitch 0.75; and (d) 4×2.5 mm, pitch 1.5.

For GE scanners, a channel width setting equal to half of the image thickness was sufficient to produce artifact‐free images for pitch values close to 1. For GE LS RT, 4×1.25 mm and a pitch of 0.75 Fig. 2(a) offered the best image quality for 2.5 mm thick images, while 4×2.5 mm and 1.5 pitch Fig. 2(d) settings caused severe helical artifacts. There was no substantial difference in the severity of artifacts for the settings 4×1.25 mm and pitch 1.5 Fig. 2(b), and 4×2.5 mm and pitch 0.75 Fig. 2(c), though the former produced better low‐contrast resolution.

**Figure 3:**
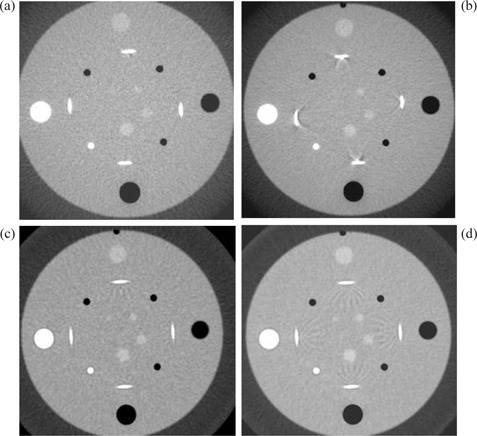
Artifact evaluation of 3 mm thick images of the Catphan phantom for Philips MX8000: (a) 16×0.75 mm, pitch 0.688; (b) 16×0.75 mm, pitch 1.438; (c) 16×1.5 mm, pitch 0.688; (d) 16×1.5 mm, pitch 1.313. Note: Windows and levels were adjusted separately for optimal display.

**Figure 4:**
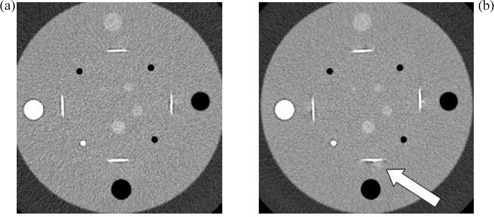
Artifact evaluation of 3 mm thick images of the Catphan phantom for Philips AcQsim single‐channel CT. (a) pitch 1.0; (b) pitch 1.5.

The Philips CT scanners behaved quite differently from the GE CT scanners (Fig. 3). For the Philips MX8000, 16×1.5 mm detector configuration for 3 mm image thickness setting (i.e., a channel width equal to half image thickness) caused severe artifacts for all available pitches Fig. 3(D). A channel width of 1/4 of the image thickness with pitch less than 1 was needed to eliminate helical artifacts Fig. 3(a) on this scanner. The other two settings, 16×0.75 mm, pitch 1.438 Fig. 3(b) and 16×1.5 mm, pitch 0.688 Fig. 3(c), resulted in less severe and different artifacts; the setting for Fig. 3c were better than those for Fig. 3b. For Philips AcQsim CT, helical artifacts increased with pitch (Fig. 4).

**Figure 5:**
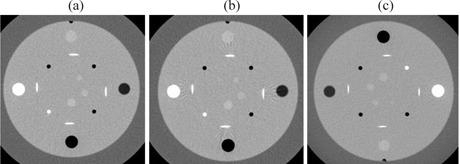
GE 16‐channel CT comparison, 2.5 mm thick images, for detector configuration: 16×1.25 mm. GE LS RT16, pitch 0.562 (a), pitch 1.375 (b), and GE LS16, pitch 1.375 (c).

Helical artifacts appeared to be scanner/model specific, not only different across manufacturers, but also different across models made by a single manufacturer. In Fig.5, we compared the images from GE LS RT16 Fig. 5(a), 5(b) with those from GE LS16 Fig. 5(c). For the same pitch (1.375) and detector configuration (16×1.25 mm), the GE LS RT16 produced more severe artifacts than the GE LS16 Fig. 5(b), 5(c). The combinations of the largest channel width and largest pitch that produced artifact‐free images for all scanners are listed in Table 1.

**Table 1:** Combination of the largest channel width and largest pitch that produced artifact‐free images.

*Model*	*Image thickness (mm)*	*Pitch*	*Detector configuration (mm)*	*Head* ${\text{CTDI}}_{W}/100\text{mAs}$ *(mGy)*	*Body* ${\text{CTDI}}_{W}/100\text{mAs}$ *(mGy)*
Philips MX8000	3	0.688	16×0.75=12	13.2	4.2
Philips AcQsim	3	1	1×3.00=3	13.0	3.5
GE LS16	2.5	1.375	16×1.25=20	14.4	7.3
GE LS RT16	2.5	0.938	16×1.25=20	12.4	6.8
GE LS RT	2.5	0.75	4×1.25=5	23.8	10.4
	*2.5*	*0.75*	4×2.5=10 ^*^	*17.7*	*6.4*

* Not artifact‐free for helical scan, but for dose comparison.

The helical streak artifact appears to depend on the combination of detector configuration and pitch, and that the relationships between these characteristics are not consistent between or even within vendors. The severity of artifacts increased with increasing pitch. The artifact evaluation in Tables 2, 3 and 4 based on the high‐contrast section of the Catphan phantom gives additional information for specific scanner settings.

**Table 2:** Image quality results for GE LS RT16.

*Rotation Time (s)*	*Mode / Pitch*	*High Contrast Resolution (lp/cm)*	*1% Low Contrast Diameter (mm)*	*Artifact Present?*
0.5	Axial	7	4	No
1.0	Axial	7	4.5	No
0.5	0.562	7	4	No
0.5	0.938	7	4	No
0.8	0.938	8	4	No
0.5	1.375	7	4.5	Yes
0.8	1.375	7	4	Yes

Technique: effective mAs=240, image thickness 2.5 mm, detector configuration 16×1.25 mm. Low contrast was defined as the diameter of the smallest circular target detectable.

**Table 3:** Image quality results for GE LS16.

*Rotation Time (s)*	*Pitch*	*High Contrast Resolution (lp/cm)*	*1% Low Contrast Diameter (mm)*	*Artifact Present?*
0.5	0.562	8	3.5	No
0.8	0.562	8	4	No
0.8	0.938	7	4	No
0.8	1.375	7.5	3.5	Slight
1	1.75	7	3	Yes

Technique: effective mAs=240, image thickness 2.5 mm, detector configuration 16×1.25 mm. Low contrast was defined as the diameter of the smallest circular target detectable.

**Table 4:** Image quality results for Philips MX8000.

*Rotation Time (s)*	*Pitch*	*High Contrast 1% Resolution (lp/cm)*	*Low Contrast Diameter (mm)*	*Artifact Present?*
0.4	0.564	8	4	No
0.5	0.563	8	3.5	No
0.75	0.563	8	3	No
1.5	0.563	8	3	No

Technique: effective mAs=240, image thickness 3 mm, detector configuration 16×0.75 mm. Low contrast was defined as the diameter of the smallest circular target detectable.

### B. Resolution evaluation

**Figure 6:**
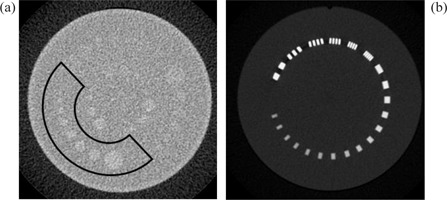
Sample image sections used for resolution evaluation: (a) Catphan CTP515 mode, the marked area is 1% contrast which was used for low‐contrast resolution evaluation; (b) Catphan CTP528 mode, use for high‐contrast resolution evaluation. Techniques: GE LightSpeed RT16, pitch 0.938, rotation time 0.8 sec, 16×1.25 mm detector configuration, 2.5 mm image thickness (display not optimized).

Image resolution (low and high contrast) was evaluated using the phantom sections shown in Fig. 6. For these GE scanners (Tables 2 and 3), the image resolution did not appear to be affected by pitch and rotation time, provided that mA was adjusted appropriately. For high‐contrast resolution, the variation was within 1 line pair (lp) /cm around the average value of 7.3lp/cm. For low contrast resolution, the variation was within 0.6 mm over the average diameter of 3.9 mm for 1% detectable low‐contrast objects. These variations may be due to quantum and statistical fluctuations. However, GE LS16 had systematically better high‐ and low‐contrast resolution than GE LS RT16. GE LS16 has a maximum tube current limit of 440 mA, thus a pitch of less than 1 had to be used to achieve 240 effective mAs for 0.5s gantry rotation. Of these two GE models, the larger bore CT scanner had lower image quality and was more prone to helical artifacts.

A similar approach was used for images obtained using Philips MX8000 (Table 4). A clear trend was observed that short rotation time (0.5 sec or less) caused slight low‐contrast resolution degradation, though high‐contrast resolution remained unaffected. The image resolution was best for Philips MX8000, followed by GE LS16, and GE RT16.

### C. Radiation Dosimetry

The CTDIW was normalized for 100 mAs using the standard head and body phantoms ^(11)^ and represents the most recent annual dosimetry evaluations. CTDIW / 100 mAs varied by nearly a factor of 2 from 12.4 mGy to 23.8 mGy among the five scanners for the head phantom. For the head CTDI phantom, normalized dose (CTDIW / 100 mAs) was highest for GE RT, followed by GE LS16, Philips MX8000, and Philips AcQsim, and was lowest for GE LS RT16.

For the body CTDI phantom, CTDIW / 100 mAs ranged from 3.5−10.4mGy; this value was highest for GE LS RT, followed by GE LS16, GE LS RT16, and Philips MX8000, and was lowest for Philips AcQsim. For this study, the head CTDI dose results would be more relevant than the body CTDI values. The normalized body CTDI results were included for completeness.

## IV. DISCUSSION

We found that for GE scanners, a channel width setting equal to half of the image thickness was sufficient to produce artifact‐free and good quality images for pitch values close to 1. Our result with GE RT 4‐channel CT agrees with Takahashi, et al.^(3)^ who determined that for this scanner model, the settings that achieved the best IQ were found to be 4×1.25 mm detector configuration and a pitch of 0.75 (though that study was only focused on depicting arterial stenosis, and imaging artifact was not specifically evaluated).

For GE RT, the detector setting 4×1.25 mm resulted in a high CTDIW of 2.38 cGy (Table 1), while 4×2.5 mm setting lowered CTDIW to 1.77cGy due to improved dose efficiency. If a faster scan speed is needed, we would suggest using 4×y2.5 mm and pitch 0.75 rather than 4×1.25 mm and pitch 1.5, though they have the same table speed and similar image quality.

For Philips MX8000, a channel width of 1/4 of the image thickness with a pitch of less than 1 was needed to eliminate helical artifacts. However, the MX8000 had overall somewhat better high‐ and low‐contrast resolution than GE scanners (Tables 2, 3, 4). Differences between these two manufacturers may be due to the different reconstruction algorithms or different dose values (matching technique parameters does not necessarily result in equivalent radiation output or quality between scanners). We observed a larger than expected difference in normalized dose (CTDIW / 100 mAs) among these scanners; when using the head CTDI phantom, there was a range of a factor of 2 in CTDIW / 100 mAs. When using the body CTDI phantom, we observed a difference between vendors by a factor of 3. In general, when spatial resolution was enhanced, artifacts present in the image were also enhanced.

In helical CT, data re‐binning and interpolation are used to generate planar transverse images from helical attenuation data. This process yields better quality images if helical data is more finely spaced. The choice of detector configuration is generally a much more powerful method of reducing helical artifact than decreasing scan pitch. Sometimes, the selection of the maximum number of active channels and the finest channel width must be balanced against the need for a faster couch speed.

The small differences in spatial resolution shown in Tables 2 to 4 are not likely to be detectable in clinical images, because the DFOV of 50 cm or greater over the 512×512 matrix used in CT simulation limits the achievable spatial resolution. Some difference in contrast resolution is expected due to the different bore sizes, detector designs and reconstruction algorithms. Increasing exposure, or effective mAs, will increase low‐contrast resolution across all scanners.

Though phantom studies are appropriate for comparative evaluations, they are not accurate simulations of patient scans. A major difference between a phantom and a patient is that a patient often moves (voluntarily or involuntarily) during a CT acquisition, especially for those of the chest and abdomen. The resulting blur is not present on the stationary phantom images. Faster rotation time reduces motion‐blurring artifact^(12)^ and improves patient image quality, though it should not affect the phantom image quality. Because fast scan time (breath‐hold) is not desired in CT simulation, a reasonably low pitch, fast rotation and fine detector configuration should be used to improve image quality and also to acquire patient images in a more averaged position rather than a snapshot of one moment. In general, we recommend using fast gantry rotation for chest and abdomen scans, 0.5−0.8 sec for example, if the options are available.

Image resolution evaluation can be used as a reference when setting up scan protocols for radiotherapy simulation. Tube current modulation should not affect the appearance of helical artifacts. However, tube current modulation may affect low‐contrast resolution, because effective mAs will vary in the x/y plane, along the axis, or both.

## V. CONCLUSIONS

In conclusion, we have quantified the effects of changes in various parameters used for RO CT simulation on specific image metrics. Given the differences we found between CT machines in our own study, choosing optimal image acquisition parameter settings for CT simulation purposes may require a phantom evaluation for each scanner model to be used.
